# Systematic review of clinical practice guidelines in the diagnosis and management of thyroid nodules and cancer

**DOI:** 10.1186/1741-7015-11-191

**Published:** 2013-08-29

**Authors:** Tsai-Wei Huang, Jun-Hung Lai, Mei-Yi Wu, Shiah-Lian Chen, Chih-Hsiung Wu, Ka-Wai Tam

**Affiliations:** 1 Department of Nursing, College of Medicine and Nursing, Hung Kuang University, Taichung, Taiwan; 2 Department of Internal Medicine, Erlin Branch of Changhua Christian Hospital, Taichung, Taiwan; 3 Division of Nephrology, Department of Internal Medicine, Taipei Medical University - Shuang Ho Hospital, Taipei, Taiwan; 4 Center for Evidence-based Health Care, Taipei Medical University - Shuang Ho Hospital, Taipei, Taiwan; 5 Department of Surgery, School of Medicine, College of Medicine, Taipei Medical University, Taipei, Taiwan; 6 Division of General Surgery, Department of Surgery, Taipei Medical University - Shuang Ho Hospital, Taipei, Taiwan; 7 Graduate Institute of Clinical Medicine, College of Medicine, Taipei Medical University, Taipei, Taiwan; 8 Center for Evidence-based Medicine, Taipei Medical University, Taipei, Taiwan; 9 Evidence-based Medicine Center, Taipei Medical University Hospital, 252 Wu-Hsing Street, Taipei 11031, Taiwan

**Keywords:** Clinical practice guidelines, Thyroid cancer, Thyroid nodule, Systematic review

## Abstract

**Background:**

Given the uncertainties regarding thyroid nodule assessment and management, physicians require systematically and transparently developed recommendations. This systematic review assesses the quality and consistency of the recommendations of international clinical practice guidelines (CPGs) for the diagnosis and management of thyroid nodules and cancer to assist physicians in making appropriate recommendations.

**Methods:**

The CPGs on the management of thyroid nodules and cancer published before June 2013 were retrieved. All the reviewed guidelines were in English. Four reviewers independently assessed the rigor of guideline development by using the Appraisal of Guidelines Research and Evaluation II (AGREE-II) instrument, and their reported evidence was evaluated.

**Results:**

Ten eligible guidelines were included: nine had been developed by professional organizations, and the remaining guideline was endorsed by an independent regional body. Three guidelines achieved a score of greater than 50% in all six AGREE-II domains. Guidelines scored highest on the measurement of ‘scope and purpose’ (≥61.1% for eight CPGs) and lowest on the measurement of ‘applicability’ (≤38.5% for five CPGs). The overall quality ranged from 3.0 to 6.25 on a seven-point scale on the AGREE-II tool. Most CPG recommendations on the management of thyroid cancer were relatively consistent. Guidelines varied regarding the indication of fine-needle aspiration for thyroid nodules, as well as in their suggestions for postoperative radioiodine ablation.

**Conclusions:**

Our analysis showed that the current CPGs varied in methodological quality. More effort is needed to improve the quality of recommendations on the diagnosis and management of thyroid nodules and cancer.

## Background

Thyroid nodules are a common clinical problem. They are more common in women, and their incidence increases with age. Epidemiologic studies have shown the prevalence of palpable thyroid nodules in iodine-sufficient parts of the world to be approximately 5% in women and 1% in men [1,2]. Thyroid nodules are clinically important because they can indicate thyroid cancer, which occurs in 5% to 15% of the population, depending on age, sex, history of radiation exposure, family history, and other factors [3,4]. Thyroid cancer is the most common malignant endocrine tumor, but represents approximately 1% of all malignancies [5]. Differentiated thyroid cancer (DTC), which includes papillary and follicular cancer, comprises the vast majority (90%) of all thyroid cancers [6].

The standard for the diagnosis and management of thyroid nodules and cancer is still inconclusive. Several theories and practices, including the indication of fine-needle aspiration (FNA), the role of the thyroid scan, the extension of thyroid surgery for DTC, the role of cervical lymph-node dissection, and the indication of radioiodine ablation (I^131^), are questionable. These issues need to be clearly addressed by valid, reliable, independent, and easily applicable clinical practice guidelines (CPGs). Several notable organizations have developed guidelines containing recommendations for thyroid nodules and cancer management. However, guidelines on the same topic can conflict with each other, and the quality and independence of the guidelines are of concern. Therefore, clinicians require guidelines that are systematically developed, and that provide transparent estimates of the benefits and harms of interventions [7-9].

The Appraisal of Guidelines, Research, and Evaluation (AGREE) instrument is a tool used for thoroughly assessing the quality of guidelines [10]. The original AGREE instrument was published in 2003 by a group of international guideline developers and researchers, the AGREE Collaboration. The updated version, the AGREE-II instrument, was released in 2010 and was funded by the Canadian Institutes of Health Research [11]. AGREE has become the standard in the evaluation and development of CPGs [12,13]. Using the AGREE-II instrument, we systematically reviewed and assessed the quality and consistency of the recommendations of CPGs on the diagnosis and management of thyroid nodules and cancer.

## Methods

### Selection criteria

We selected CPGs that provided recommendations on the diagnosis and management of thyroid nodules or cancer. For inclusion in our study, the guidelines were required to (1) have published in English, and (2) examine all subgroups of the population to ensure that the CPGs catered for the needs of those with comorbidities in various settings. When more than one set of guidelines was produced by the same professional body, only the most recently issued was considered. We excluded guidelines that (1) focused exclusively on thyroid disease among special groups (for example, anaplastic thyroid cancer, pregnant women or children); (2) focused entirely on a unique technique, such as the procedure guideline for radioiodine therapy; (3) concentrated on a non-nodular disease, such as thyroid dysfunction; (4) contained recommendations for other diseases, such as neuroendocrine tumors or head and neck cancer; or (5) reported non-original recommendations (referring to other sets of guidelines).

### Search strategy and guideline selection

Two reviewers (K-WT and T-WH) searched for relevant studies using keyword searches of the following electronic databases: MEDLINE, EMBASE, CINAHL, the National Guideline Clearinghouse, the National Institute for Health and Clinical Excellence, the Scottish Intercollegiate Guidelines Network (SIGN), and the Guidelines International Network (G-I-N) International Guideline Library. The following terms and Boolean operators were used in MeSH and free-text searches: thyroid, cancer OR carcinoma OR neoplasm, nodule OR mass OR tumor, and guidelines OR recommendations. The ‘related articles’ facility in PubMed was used to broaden the search. The last search was performed in June 2013.

### Recommendation extraction and analysis

Two reviewers (K-WT and T-WH) independently extracted the details of the guidelines pertaining to the CPG characteristics (for example, country or region, year of dissemination, development team, and funding organization), the goals of the guidelines, the target population and audience, the recommendations related to the diagnosis of thyroid nodules, the recommendations related to the management of thyroid nodules and cancer, and the evaluation of options for postoperative follow-up. The individually recorded decisions of two reviewers were compared, and any disagreement was resolved based on the evaluation of a third reviewer (J-HL).

We constructed a table to compare the recommendations from the selected guidelines. The table was divided into the following sections and items, based on the types of clinical practices that focus on thyroid nodules and cancer: (1) diagnosis: an indication of FNA, the role of routine serum calcitonin, and an indication of a thyroid scan; (2) treatment: an indication of total thyroidectomy for DTC, and the role of cervical lymph node dissection in node-negative patients; and (3) postoperative care: an indication of I^131^ ablation, and a target level of thyroid-stimulating hormone (TSH) suppression therapy.

### Guideline quality assessment

Four investigators (K-WT, T-WH, J-HL and M-YW) independently appraised all the selected guidelines by using the AGREE-II instrument [10]. AGREE-II consists of 23 key items organized into 6 domains: (1) ‘scope and purpose’thinsp;, (2) ‘stakeholder involvement’thinsp;, (3) ‘rigor of development’thinsp;, (4) ‘clarity and presentation’thinsp;, (5) ‘applicability’thinsp;, and (6) ‘editorial independence’. Each domain captured a separate dimension of the guideline quality with a seven-point scale (from 7 (strongly agree) down to 1 (strongly disagree)). For each reviewer, AGREE-II scores were calculated as a percentage by using the sum of the seven-point scale and the maximum possible score (range 0% to 100%). Item scores were discussed by the four reviewers, and large scoring discrepancies (defined as ≤3 points difference in the score assigned by the appraisers to the same item) were resolved by consensus. We considered satisfactory any guideline that scored at least 50% in all six domains, as defined by AGREE-II. Upon completing the 23 items, each reviewer provided an overall assessment of the guideline. We compared the mean values of each of the six domain scores and the overall scores obtained by the four reviewers to evaluate the possible risk of bias and the recommendation for future use for each CPG appraised.

## Results

### Literature search

**Figure 1:**
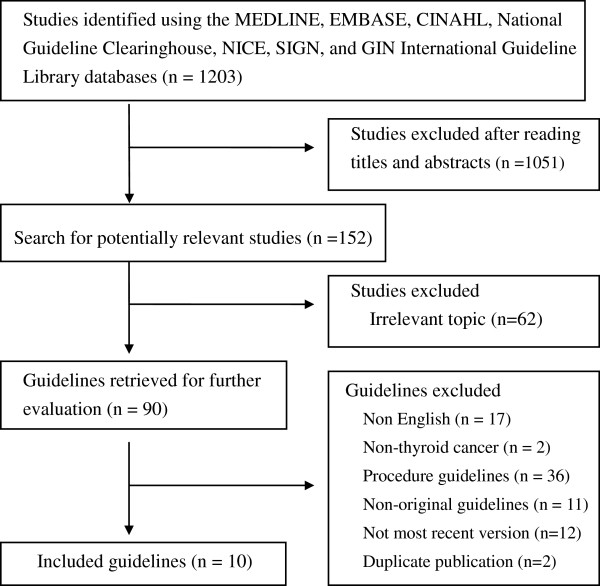
Flowchart of the selected clinical practice guidelines.

The flowchart in Figure 1 shows the process by which we screened and selected the guidelines. Our initial search yielded 1,203 citations, of which 1,051 were deemed ineligible by screening their titles and abstracts, and 62 were excluded due to irrelevant topic. This left 90 eligible studies. Of these, 79 reports were excluded from our final analysis for the following reasons: 17 were non-English guidelines, 2 were concentrated on non-thyroid cancer, 36 were the procedure guidelines, 12 guidelines were not the most recent version, 2 being duplicate publication, and 11 were the comment of original guidelines. The remaining ten eligible guidelines were included in our analysis [14-23].

### Characteristics of the guidelines

Table 1 presents a summary of the characteristics of the selected guidelines. The ten guidelines were published between 2000 and 2013. Of the ten guidelines, four were new [19,20,22,23], and the rest were updates. Of the selected guidelines, 3 were developed in multiple countries: the Latin American Thyroid Society (LATS) used the guidelines from 13 Latin American countries [20], the European Society for Medical Oncology (ESMO) was approved in Europe [17], and American Association of Clinical Endocrinologists, Associazione Medici Endocrinologi, and European Thyroid Association (AACE/AME/ETA) was contributed by the USA and Europe [14]. The remaining CPGs were conducted in the USA [15,21], the UK [16,22], Germany [18], The Netherlands [19], and Spain [23]. Nine guidelines were produced by independent professional scientific organizations, and the remaining guideline was developed with the endorsement of a regional independent body [22]. Five CPGs were funded by independently professional organizations [14-16,19,21], and the remainder did not disclose a funding source.

**Table 1:** Characteristics of the thyroid nodule management guidelines included in the study

**Guidelines (Year)**	**Organization**	**Country or region**	**Level of development**	**Status**	**Funding**
AACE/AME/ETA (2010)	American Association of Clinical Endocrinologists, Associazione Medici Endocrinologi, and European Thyroid Association	USA, Italy, and Europe	Professional organization	Updated	American Association of Clinical Endocrinologists
ATA (2009)	American Thyroid Association	USA	Professional organization	Updated	American Thyroid Association
BTA (2007)	British Thyroid Association, and Royal College of Physicians	UK	Professional organization	Updated	British Thyroid Association
ESMO (2012)	European Society for Medical Oncology	Europe	Professional organization	Updated	Not available
GAES (2013)	German Association of Endocrine Surgeons	Germany	Professional organization	Updated	Not available
IKNL (2007)	Dutch Endocrine Society, and Dutch Society of Nuclear Medicine	The Netherlands	Professional organization	New	Dutch Society of Medical Specialists
LATS (2009)	Latin American Thyroid Society	Latin America	Professional organization	New	Not available
NCCN (2013)	National Comprehensive Cancer Network	USA	Professional organization	Updated	NCCN Foundation
NCN (2000)	Northern Cancer Network	UK	Regional independent body	New	Not available
SEOM (2011)	Spanish Society of Medical Oncology	Spain	Professional organization	New	Not available

### Appraisal of guidelines

With the exception of the ESMO and Spanish Society of Medical Oncology (SEOM) CPGs [17,23], all the selected guidelines stated the methods used in the literature search, the quality of the evidence, and the strengths of the recommendations reported. Table 2 presents the domain scores (%) for the ten CPGs that were assessed using the AGREE-II instrument. Three of the selected guidelines performed satisfactorily, achieving a score of greater than 50% in all six AGREE-II domains [16,19,21]. The guidelines generally received the lowest scores for domain 5, ‘applicability’, among all six AGREE-II domains (≤38.5% for five CPGs). Guidelines scored highest in domain 1 (≥61.1% for eight CPGs). The British Thyroid Association (BTA) and Dutch Endocrine Society/Dutch Society of Nuclear Medicine (IKNL) guidelines scored the highest on domain 1 (87.5% and 87.5%) and domain 2 (76.4% and 75%) [16,19]. Moreover, the IKNL guideline scored the highest for domain 3 (88.5%) [19]. The National Comprehensive Cancer Network (NCCN) guideline scored the highest for domain 4 (81.9%) and domain 6 (85.4%) [21]. Domain 5 scored consistently low across the CPGs.

**Table 2:** Domain scores (%) of the ten clinical practice guidelines assessed using the AGREE-II instrument

**Domain**	**AACE/AME/ETA (2010)**	**ATA (2009)**	**BTA (2007)**	**ESMO (2012)**	**GAES (2013)**	**IKNL (2007)**	**LATS (2009)**	**NCCN (2013)**	**NCN (2000)**	**SEOM (2011)**
Domain 1: scope and purpose	76.4	84.7	87.5	33.3	61.1	87.5	79.2	79.2	68.1	40.2
Domain 2: stakeholder involvement	65.2	72.2	76.4	22.2	54.2	75	44.4	69.4	51.4	26.4
Domain 3: rigor of development	62.5	62.0	66.1	21.4	58.9	88.5	45.8	58.3	36.4	16.1
Domain 4: clarity of presentation	77.8	70.8	69.4	38.9	63.9	73.6	54.2	81.9	56.9	45.8
Domain 5: applicability	38.5	42.7	56.3	22.9	35.4	63.5	40.6	57.2	29.2	21.9
Domain 6: editorial independence	79.2	81.3	75	39.6	45.8	79.2	52.1	85.4	29.2	33.3

Guideline group abbreviations are as follows: *AACE/AME/ETA* American Association of Clinical Endocrinologists, Associazione Medici Endocrinologi, and European Thyroid Association, *ATA* American Thyroid Association, *BTA* British Thyroid Association and Royal College of Physicians, *ESMO* European Society for Medical Oncology, *GAES* German Association of Endocrine Surgeons, *IKNL* Dutch Endocrine Society and Dutch Society of Nuclear Medicine, *LATS* Latin American Thyroid Society, *NCCN* National Comprehensive Cancer Network, *NCN* Northern Cancer Network and *SEOM* Spanish Society of Medical Oncology.

Table 3 details the mean scores for the 23 items and the overall mean scores for each domain from the 10 guidelines evaluated as assessed with AGREE-II, by averaging the scores from each of the 4 investigators. The overall quality of the CPGs ranged from 3.00 ± 0.00 to 6.25 ± 0.50 of a possible 7 on the AGREE-II tool. In general, the lowest mean scores were for item 5 (range 1.75 ± 0.50 to 4.25 ± 1.71) and item 21 (range 2.0 ± 0.0 to 4.0 ± 0.82), which indicated that the included guidelines seldom provided the views or preferences of the target population or the monitoring or auditing criteria of the key recommendations.

**Table 3:** Quality of the thyroid nodule/cancer clinical practice guidelines for the six domains of the AGREE-II Instrument (D1 to D6) and the overall impression of the four assessors

**Domain**	**AACE/AME/ETA (2010)**	**ATA (2009)**	**BTA (2007)**	**ESMO (2012)**	**GAES (2013)**	**IKNL (2007)**	**LATS (2009)**	**NCCN (2013)**	**NCN (2000)**	**SEOM (2011)**
D1										
1	6 (0)	6.25 (0.5)	6.5 (0.58)	3.5 (0.58)	5 (0.82)	6.5 (0.58)	6 (0)	6.25 (0.5)	6 (0)	3.5 (0.58)
2	5.75 (0.5)	6.5 (0.58)	6.25 (0.96)	3.25 (0.96)	5 (0.50)	6.5 (0.58)	6 (0)	5.5 (0.58)	4.25 (0.5)	3.5 (0.58)
3	6 (0.82)	5.5 (1.29)	6 (1.41)	2.25 (1.26)	4.25 (0.96)	5.75 (1.5)	5.25 (0.96)	5.5 (1.29)	5 (0.82)	3.25 (0.5)
D2										
4	5.5 (0.58)	6.25 (0.5)	6.25 (0.5)	2.5 (1.29)	5 (1.15)	7 (0)	4 (1.41)	6.75 (0.5)	5.25 (0.5)	2.75 (0.5)
5	3.25 (0.96)	3.75 (1.71)	4.25 (1.71)	1.75 (0.50)	2.75 (0.96)	3 (1.41)	1.75 (0.50)	2.5 (0.58)	1.75 (0.5)	2 (0)
6	6 (0)	6 (0.82)	6.25 (0.5)	2.75 (0.50)	5 (0)	6.5 (0.58)	5.25 (0.50)	6.25 (0.5)	5.25 (0.96)	3 (0.82)
D3										
7	6 (0.82)	3.75 (0.96)	4.5 (1.29)	1.25 (0.50)	5 (0.82)	6.75 (0.50)	3.75 (0.96)	3.5 (0.58)	2.75 (0.50)	1.75 (0.50)
8	4 (0.82)	5.5 (1.29)	4 (0.82)	1 (0)	5.25 (0.50)	6.75 (0.50)	3 (0.82)	4.25 (0.50)	3.25 (0.50)	1.25 (0.50)
9	4.5 (1.73)	4 (1.15)	4.75 (1.50)	1.5 (0.58)	3.5 (1.29)	6.25 (0.50)	3 (0.82)	4.5 (1.29)	3.25 (0.50)	1.75 (0.50)
10	5.25 (0.96)	4.75 (0.5)	5 (1.41)	3 (0)	5.75 (0.50)	6.5 (0.58)	4.25 (1.50)	5 (0.82)	3.75 (1.50)	2 (0)
11	4.25 (1.50)	4.25 (0.96)	4.5 (1.29)	2.75 (0.96)	4.5 (0.58)	6.25 (0.50)	3.75 (1.50)	5.75 (0.50)	3.25 (1.26)	2.25 (0.50)
12	6 (0)	5.25 (1.71)	6.25 (0.96)	4.75 (0.50)	4.5 (1.00)	6.5 (0.58)	2.75 (0.50)	4 (0.82)	2.5 (1.00)	2.75 (0.96)
13	4 (1.15)	5.5 (0.58)	5.75 (0.50)	2 (0)	4 (1.41)	5.75 (0.50)	5.25 (0.50)	4 (1.15)	4.5 (1.29)	2 (0)
14	4 (1.83)	4.75 (0.96)	5 (0.82)	2 (0)	3.75 (0.50)	5.75 (0.50)	4.25 (1.50)	5 (1.41)	2.25 (0.50)	2 (0)
D4										
15	6 (0.82)	6 (0)	6 (0)	3.75 (0.96)	5 (0)	6.25 (0.50)	4.5 (0.58)	6.25 (0.50)	4.5 (0.58)	4 (0)
16	5 (1.83)	3.75 (0.96)	3.75 (0.96)	3 (0.82)	3.75 (0.96)	4.5 (1.73)	3.75 (1.50)	5.5 (0.58)	4 (1.15)	3.25 (0.96)
17	6 (0.82)	6 (0)	5.75 (0.50)	3.75 (0.50)	6 (0)	6.25 (0.50)	4.5 (0.58)	6 (0)	4.75 (0.50)	4 (0)
D5										
18	3 (0.82)	3.25 (0.50)	4.5 (0.58)	2 (0)	2.75 (1.26)	5.25 (0.50)	3 (0)	4 (0.82)	2.75 (0.50)	2 (0)
19	4.25 (1.50)	4 (1.41)	6 (0.82)	2.75 (0.96)	4.25 (1.50)	6 (0)	4.25 (1.50)	5.75 (0.50)	3.5 (1.29)	3 (1.15)
20	3.25 (1.71)	3.75 (1.26)	3.25 (1.26)	2 (0)	3 (1.41)	4.25 (1.26)	3.5 (1.29)	4 (0.82)	2.25 (0.50)	2.25 (0.50)
21	2.75 (0.96)	3.25 (1.50)	3.75 (1.71)	2.75 (1.50)	2.5 (1.00)	3.75 (0.96)	3 (1.15)	4 (0.82)	2.5 (1.00)	2 (0)
D6										
22	5 (1.15)	6 (0.82)	5.5 (1.73)	3 (0.82)	5.25 (0.96)	6 (0)	4 (1.41)	5.75 (0.50)	1.75 (0.50)	4.5 (1.29)
23	6.5 (0.58)	5.75 (1.89)	5.5 (1.73)	3.75 (0.96)	2.25 (0.50)	5.5 (1.29)	4.25 (1.50)	6.5 (0.58)	3.75 (0.96)	1.5 (0.58)
Overall	5.75 (0.50)	6 (0)	6.25 (0.50)	3.75 (0.96)	5 (0)	6.25 (0.50)	4.25 (0.50)	5.5 (0.58)	4.25 (0.96)	3 (0)

Domain areas are as follows: D1, scope and purpose; D2, stakeholder involvement; D3, rigor of involvement; D4, clarity of presentation; D5, applicability; D6, editorial independence. All the 23 items of the Appraisal of Guidelines Research and Evaluation II (AGREE-II) instrument are rated on a seven-point scale from 7 (strongly agree) down to 1 (strongly disagree). Overall: the numbers in the overall row represent the averages of the scoring performed by four assessors. Guideline group abbreviations are as follows: *AACE/AME/ETA* American Association of Clinical Endocrinologists, Associazione Medici Endocrinologi and European Thyroid Association, *ATA* American Thyroid Association, *BTA* British Thyroid Association and Royal College of Physicians, *ESMO* European Society for Medical Oncology, *GAES* German Association of Endocrine Surgeons, *IKNL* Dutch Endocrine Society and Dutch Society of Nuclear Medicine, *LATS* Latin American Thyroid Society, *NCCN* National Comprehensive Cancer Network, *NCN* Northern Cancer Network and *SEOM* Spanish Society of Medical Oncology.

### Clinical practice guideline recommendations

Recommendations for the diagnosis and management of thyroid nodules and cancer extracted from these guidelines are shown in Table 4. Regarding the diagnostic procedures, all guidelines advocated thyroid sonography. In addition, a measurement of TSH and free thyroxine levels should be performed in all patients. A routine measurement of serum thyroidglobulin (Tg) for the initial evaluation of thyroid nodules is not recommended. However, although all the guidelines supported FNA as the procedure of choice in the evaluation of solid thyroid nodules, the CPGs define various nodular sizes as indications for FNA. The German Association of Endocrine Surgeons (GAES), IKNL, and Northern Cancer Network (NCN) guidelines recommend that FNA should be performed in all nodules [18,19,22], two CPGs recommend that the indication of FNA must be performed in nodules >0.5 cm in diameter [15,16], and the other three CPGs suggest that FNA cytology is needed when the thyroid nodule is bigger than 1 cm in the absence of clinical suspicion [14,17,23]. In the 2013 version of the NCCN guidelines, the threshold for FNA is a solid thyroid nodule >1.5 cm, if no suspicious sonographic features are present [21]. For laboratory evaluation, four CPGs recommend routine serum calcitonin, particularly before surgery for nodular thyroid disease [17-19,23]. Four guidelines suggest a serum calcitonin assay as an optional test [14,20-22], but the American Thyroid Association (ATA) guidelines cannot recommend or discourage the routine measurement of serum calcitonin, because of insufficient evidence [15]. For radionuclide scanning, three guidelines mentioned thyroid scintigraphy in a single thyroid nodule with a low TSH level [14,15,21], and the GAES CPGs recommend thyroid scintigraphy before the planning and execution of an operation [18].

**Table 4:** Recommendations stated in the ten clinical practice guidelines

**Recommendations**	**AACE/AME/ETA (2010)**	**ATA (2009)**	**BTA (2007)**	**ESMO (2012)**	**GAES (2013)**	**IKNL (2007)**	**LATS (2009)**	**NCCN (2013)**	**NCN (2000)**	**SEOM (2011)**
Diagnosis										
Indications of fine-needle aspiration (without suspicions)	n >1 cm	n >0.5 cm	n >0.5 cm	n >1 cm	All nodules	All nodules	N/A	n >1.5 cm	All nodules	n >1 cm
Routine serum calcitonin	Optional	NR	N/A	R	R	R	Optional	Optional	Optional	R
Thyroid scan	Low TSH	Follicular lesion with low TSH	N/A	Unclear	Before operation	NR	N/A	Follicular lesion with low TSH	Unclear	NR
Treatment										
Indication of total thyroidectomy for DTC	N/A	n >1 cm	n >1 cm	n >1 cm	n >1 cm	n >1 cm	All sizes	n >4 cm	n >1 cm	n >4 cm
Cervical lymph node dissection (node negative)	N/A	n >4 cm	n >4 cm/male/age >45 years	Optional	Optional	N/A	n >4 cm	Optional	Unclear	Optional
Postoperative care										
Indication of I^131^ ablation	N/A	n >4 cm/high-risk patients	High-risk patients	n >2 cm/high-risk patients	N/A	High-risk patients	High-risk patients	Tg >1 ng/ml/high-risk patients	n >1 cm	High-risk patients
Target level of TSH suppression therapy (mU/L)										
High risk	N/A	<0.1	<0.1	<0.1	N/A	<0.1	<0.1	<0.1	0.01 to 0.1	<0.1
Low risk	N/A	0.1 to 0.5	0.1 to 0.5	WNL	N/A		0.4 to 1.0	Close to the lower limit		<0.1 to 0.5

Guideline group abbreviations are as follows: *AACE/AME/ETA* American Association of Clinical Endocrinologists, Associazione Medici Endocrinologi and European Thyroid Association, *ATA* American Thyroid Association, *BTA* British Thyroid Association and Royal College of Physicians, *ESMO* European Society for Medical Oncology, *GAES* German Association of Endocrine Surgeons, *IKNL* Dutch Endocrine Society and Dutch Society of Nuclear Medicine, *LATS* Latin American Thyroid Society, *NCCN* National Comprehensive Cancer Network, *NCN* Northern Cancer Network and *SEOM* Spanish Society of Medical Oncology.

*DTC* differentiated thyroid cancer, *I* iodine, *n* nodule, *N/A* not available, *NR* not recommended, *R* recommended, *Tg* thyroglobulin, *TSH* thyroid stimulating hormone, *WNL* within normal limit.

Concerning thyroid surgery for DTC, patients with node-negative cancer 1 cm in diameter or more should be treated with total or near-total thyroidectomy, except where contraindications prevent this surgery [15-19,22]. However, the NCCN and SEOM CPGs recommend total or subtotal thyroidectomy in tumors >4 cm in diameter [21,23]. In contrast, the LATS guidelines recommend total thyroidectomy for DTC, regardless of tumor size [20]. An additional surgical consideration is cervical lymph node dissection for patients with DTC. Three CPGs recommend that prophylactic central-compartment neck dissection may be performed in patients who present with a DTC with clinically uninvolved central-neck lymph nodes, especially for advanced primary tumors (nodule >4 cm) [15,16,20].

Recommendations for the postoperative care of DTC are varied. First, radioiodine ablation is recommended for all high-risk patients, as determined from the presence of metastases, incomplete excision, gross extrathyroidal extension of the tumor regardless of tumor size, or a primary tumor >4 cm, even in the absence of other higher-risk factors [15]. However, the NCN guidelines mention that routine postoperative I^131^ ablation of thyroid remnants has been shown to reduce local recurrence and improve patient survival when they have tumors more than 1 cm in diameter [22]. Moreover, the NCCN guidelines recommend that I^131^ ablation be performed in patients with thyroglobulin >1 ng/ml [21]. Second, all CPGs emphasized the need for TSH suppression therapy following DTC surgery. However, the target levels of the TSH varied across the guidelines. Initial TSH suppression to below 0.1 mU/L is recommended for high-risk patients [15-17,19-21,23], whereas NCN guidelines suggest the maintenance of the TSH at 0.01 to 0.1 mU/L [22]. Moreover, CPG recommendations were inconsistent regarding the maintenance of the TSH in low-risk patients.

## Discussion

This study assessed the quality and consistency of the recommendations of international CPGs on the diagnosis and management of thyroid nodules and cancer to assist physicians in considering the appropriate recommendations. We identified ten guidelines involving thyroid nodules and cancer management, three of which had been published between 2000 and 2007 [16,19,22]. As a general rule, CPGs should be reassessed for validity every 3 years [24,25]. Therefore, one of the CPGs reviewed here are likely to be outdated because they have not been updated in over 10 years [25]. A distinct variation in the applicability and transparency of funding sources was found among the guidelines. After applying the AGREE-II instrument to the ten guidelines, we found that guidelines developed by the BTA [16], the IKNL [19], and the NCCN scored above 50% in all six domains [21]. Moreover, the view of the target population (domain 5) on guideline development was inadequate in all ten guidelines. We found differences among guidelines with respect to the indication of FNA in low-suspicion nodules, the routine measurement of serum calcitonin, and the role of cervical lymph node dissection in node-negative patients.

The application of AGREE-II allows for an evaluation of the various aspects of guidelines. We measured the development methods of the guidelines by using the AGREE-II instrument, based on the rationale that a high methodological quality is fundamental for the integrity, reproducibility, and transparency of guidelines. Our study showed that the methodological quality of the guidelines was optimal for ‘scope and purpose’ and ‘clarity and presentation’, but received the lowest scores for ‘applicability’. Most guidelines lacked explicit statements on whether the patients’ views and preferences had been sought (item 5), whether the various options for management of the condition were clearly presented (item 16), whether the potential cost implications of applying the recommendations were considered (item 20), and on key review criteria for monitoring and/or auditing purposes (item 21). Although the AGREE-II instrument provides six independent scores for six corresponding aspects of the guidelines, we believe that clinicians would be more concerned about the ‘rigor of development’. However, the quality of ‘applicability’ domain also plays a critical role in implementation of the guideline. For a guideline to be effective, it should provide advice as to how the recommendations can be implemented, it should present a discussion of the potential impact of recommendations on resources, and it requires clearly defined criteria derived from the key recommendations. Therefore, we recommend that clinicians rely preferentially on the guidelines that performed better regarding the ‘applicability’ domain [16,19,21].

The AGREE-II instrument is used to establish a universal standard for the rigor and transparency of guideline development, and to suggest how to improve existing guidelines [11]. However, some limitations exist. One serious limitation concerns conflicts of interest. The AGREE-II instrument advocates that guidelines always report clearly, irrespective of whether conflicts exist, but several of the guidelines lacked statements about conflicts of interest. The use of a guideline-adaptation framework such as ADAPTE should be considered to develop high-quality CPGs in the future [26].

In general, the recommendations of the CPGs on the diagnosis, management, and postoperative care of thyroid nodules and cancer were consistent, despite the discrepancies between scores for the ‘rigor of development’. All guidelines advocated thyroid sonography, as well as measured the TSH and free thyroxine levels in all patients. However, no firm recommendations were made for the routine assessment of serum calcitonin and the indication of thyroid scintigraphy. Similarly, major differences across the CPGs were related to the indication of radioiodine ablation and the optimal level of TSH suppression therapy in patients with DTC (Table 4). Such situations revealed that, even when CPG developers claimed to have paired their grade of recommendations with the level of evidence, recommendations were not graded or were inconsistent. This variation may be related to the developers’ search strategy, the process of selecting scientific evidence, and the way the recommendations had been formulated [27,28].

In prospective trials, conclusions regarding the optimal selection of treatments must be based on retrospective analysis and the consensus of expert opinions [15-17,29]. Several CPGs recommend total thyroidectomy if the primary tumor is at least 1 cm in diameter or if extrathyroidal extension or metastases are present [15-18,22], but some guidelines advise that total thyroidectomy may be performed in patients with large tumors (>4 cm) in the absence of clinical suspicion [21,23]. Whereas some CPGs recommend considering routine central-neck dissection for most patients with papillary thyroid cancer [15,16,20], the guidelines from the NCCN recommend only central-neck dissection in the presence of grossly positive metastasis [16,19,21].

The strengths of our review included a comprehensive search for eligible guidelines, the systemic and explicit application of eligibility criteria, the careful consideration of guideline quality by using the AGREE-II instrument, and a rigorous analytical approach. Therefore, this study can be of additional value to already available guideline compendia and libraries such as the National Guideline Clearinghouse and the National Institute for Health and Clinical Excellence because these libraries depend on submissions from guideline organizations. However, several limitations could have biased our study. First, only CPGs written in English were included, and guidelines written entirely in other languages might have been overlooked. Second, CPGs that focus on non-DTC such as medullary thyroid cancer or unique techniques such as procedural guidelines for radioiodine therapy were excluded from our study. Third, the AGREE-II instrument is used to evaluate the guideline as a whole, and is not intended for specific, individual recommendations. However, a global appraisal on a guideline’s construction process may reflect the strength of the individual recommendations to an extent. Finally, we used only the AGREE-II instrument in evaluating the quality of the guidelines. Other instruments such as the four-item Global Rating Scale (GRS) may also play a role in guideline assessment [30]. Although the GRS is less sensitive than AGREE-II in detecting differences in guideline quality, its items did predict outcome measures related to guideline adoption.

## Conclusions

In summary, the results of our study revealed that current CPGs varied in methodological quality, and increased efforts are required to improve the quality of recommendations on the diagnosis and management of thyroid nodules and cancer. We therefore encourage clinicians to use the guidelines from this review with higher AGREE-II rigor scores for managing patients with thyroid nodules or cancer.

## Competing interests

The authors declare they have no conflicts of interest or financial ties to disclose.

## Authors’ contributions

K-WT and T-WH devised the study. K-WT, T-WH, J-HL, and M-YW extracted, analyzed and interpreted the data. K-WT and T-WH wrote the first draft. All authors contributed to subsequent versions, and approved the final article. K-WT is the corresponding author. All authors read and approved the final manuscript.

## Pre-publication history

The pre-publication history for this paper can be accessed here:

http://www.biomedcentral.com/1741-7015/11/191/prepub
